# Supplementary material for: Coordinated crosstalk between microtubules and actin by a spectraplakin regulates lumen formation and branching
Source: eLife. 2020 Oct 28;9:e61111. doi: 10.7554/eLife.61111 (PMC7661041; doi:10.7554/eLife.61111)
Supplement: Supplementary file 1. — Description of the quantification of phenotypes from Figure 3. [file elife-61111-supp1.docx]

**SUPPLEMENTARY FILE 1**

**Coordinated crosstalk between microtubules and actin by a spectraplakin regulates lumen formation and branching**

Delia Ricolo^1,2^ and Sofia J. Araújo^1,2*^

^1^Department of Genetics, Microbiology and Statistics, School of Biology, University of Barcelona, 08028 Barcelona, Spain

^2^Institute of Biomedicine University of Barcelona (IBUB), Barcelona, Spain

^*^Corresponding author. email: S. J. Araújo sofiajaraujo@ub.edu,

**Supplementary Table 1.** DSRF-specific sequences in upstream and downstream regions of the *shot* gene. Sequences are shown within 2000 bp of the TSS with more than 70% similarity to the specific DSRF binding sequences, indicating their score and the chain of chromosome 2 in which they were found.

The DSRF-binding sequences identified, overlap with regions P1, P2, and P3. In the case of the P1 promoter, we found the sequences in the table spanning 13,926,470-13,927,188; in the case of P2, the first three DSRF binding sequences of the table are located within this region, which include 13,943,065-13,329; and in the case of P3, we found DSRF binding sequences within 13,912,773-13,912,789.

| **Chr** | **Start** | **End** | **Length** | **Score** | **Chain** | **Sequence** |
| --- | --- | --- | --- | --- | --- | --- |
| chr2R | 13,943,313 | 13,943,329 | 16 | 0.75 | + | TTTCCAAATGGGGTCA |
| chr2R | 13,943,313 | 13,943,329 | 16 | 0.75 | - | TGACCCCATTTGGAAA |
| chr2R | 13,943,065 | 13,943,081 | 16 | 0.74 | + | TCCCGATCTGTGGTCA |
| chr2R | 13,927,172 | 13,927,188 | 16 | 0.78 | + | TACTCATTTATGGACA |
| chr2R | 13,927,172 | 13,927,188 | 16 | 0.76 | - | TGTCCATAAATGAGTA |
| chr2R | 13,926,681 | 13,926,697 | 16 | 0.74 | + | TGTCGATCTGTGGTCT |
| chr2R | 13,926,470 | 13,926,486 | 16 | 0.79 | + | TGACCAAATATGAGGA |
| chr2R | 13,926,470 | 13,926,486 | 16 | 0.80 | - | TCCTCATATTTGGTCA |
| chr2R | 13,912,773 | 13,912,789 | 16 | 0.76 | + | ACTCCATATAGGGCAA |
| chr2R | 13,912,260 | 13,912,276 | 16 | 0.73 | + | TTAACATAAGTGGTCA |
| chr2R | 13,912,260 | 13,912,276 | 16 | 0.74 | - | TGACCACTTATGTTAA |
| chr2R | 13,912,773 | 13,912,789 | 16 | 0.78 | - | TTGCCCTATATGGAGT |

**Supplementary Table 2. DSRF positional weight matrix (PWM)**

Specific DSRF-binding sequences known in vertebrates (Khan et al., 2018).

**
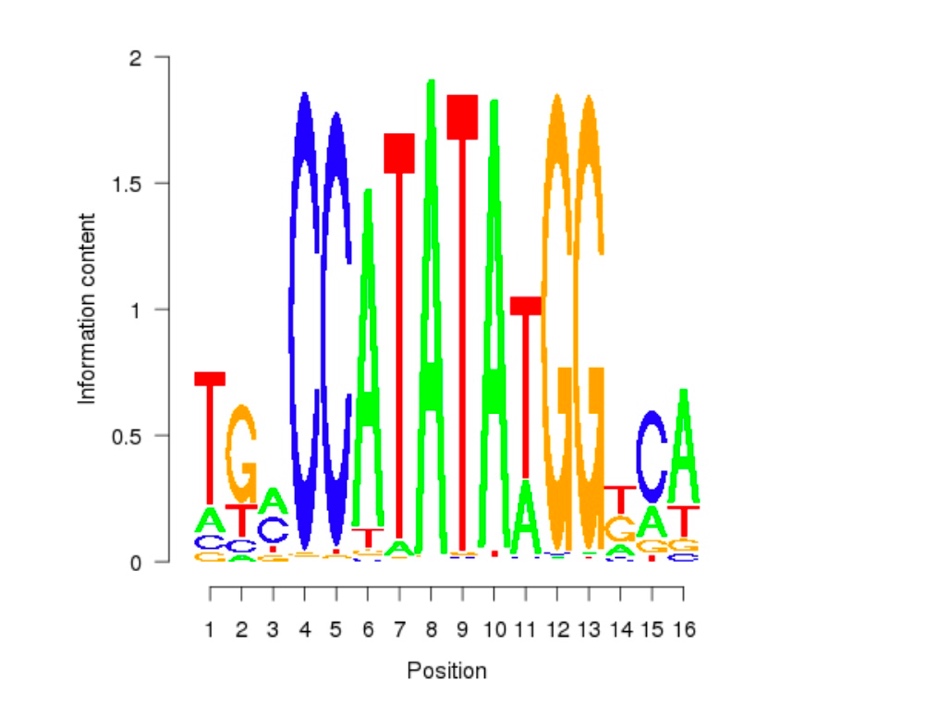
**

**Quantification of the number of bifurcations (GB TCs) per embryo of the indicated genotype** **in Figure 3**.

In the graph (panel I) embryos with 0 bifurcations are represented by grey columns, embryos with 1 bifurcation columns in light blue, 2 bifurcations columns in blue, 3/more bifurcations columns in dark blue. To better quantify the phenotypes, we subdivided *Rca1* and ShotOE embryos in groups characterized by 1, 2, “3/or more” branched TC lumina. 80% of *Rca1* mutant embryos had TCs with supernumerary lumina (n=40); in particular, 24% of embryos had 1 bifurcation, 30% of embryos had 2 bifurcations and 28% of embryos had 3 or more bifurcations. In ShotOE embryos, 57,5 % of embryos had TCs affected; 25% with one bifurcation, 22.5% with two bifurcations and 10% with three or more bifurcations (n= 40). We then analysed the phenotype in *Rca1,* ShotOE embryos (n=28). These embryos had bifurcation phenotypes in 96,3 % of the cases; 11,1% with 1 bifurcation 29,6% with 2 bifurcations and 55,6% with 3 or more bifurcations. So, in *Rca1,* ShotOE embryos, we observed a higher number of embryos with 2 or 3/more bifurcations and a lower number of embryos with 0 or 1 bifurcations in relation to *Rca1*, suggesting that the effect of *Rca1* LOF and ShotOE was additive in producing 2 or 3/more bifurcations.

**References**

Khan, A., O. Fornes, and M.G. Arnaud Stigliani, Jaime A Castro-Mondragon, Robin van der Lee, Adrien Bessy, Jeanne Chèneby, Shubhada R Kulkarni, Ge Tan, Damir Baranasic, David J Arenillas, Albin Sandelin, Klaas Vandepoele, Boris Lenhard, Benoît Ballester, Wyeth W Wasserman, François Parcy, Anthony Mathelier 2018. JASPAR 2018: update of the open-access database of transcription factor binding profiles and its web framework. *Nucleic Acids Res.* 46:D260-D266.
